# The 14-3-3 Protein Family and Schizophrenia

**DOI:** 10.3389/fnmol.2022.857495

**Published:** 2022-03-14

**Authors:** Meaghan Navarrete, Yi Zhou

**Affiliations:** Department of Biomedical Sciences, Florida State University College of Medicine, Tallahassee, FL, United States

**Keywords:** 14-3-3 proteins, schizophrenia, knockout mice, animal models, human studies, genetic linkage

## Abstract

Schizophrenia is a debilitating mental disorder that affects approximately 1% of the world population, yet the disorder is not very well understood. The genetics of schizophrenia is very heterogenous, making it hard to pinpoint specific alterations that may cause the disorder. However, there is growing evidence from human studies suggesting a link between alterations in the 14-3-3 family and schizophrenia. The 14-3-3 proteins are abundantly expressed in the brain and are involved in many important cellular processes. Knockout of 14-3-3 proteins in mice has been shown to cause molecular, structural, and behavioral alterations associated with schizophrenia. Thus, 14-3-3 animal models allow for further exploration of the relationship between 14-3-3 and schizophrenia as well as the study of schizophrenia pathology. This review considers evidence from both human and animal model studies that implicate the 14-3-3 family in schizophrenia. In addition, possible mechanisms by which alterations in 14-3-3 proteins may contribute to schizophrenia-like phenotypes such as dopaminergic, glutamatergic, and cytoskeletal dysregulations are discussed.

## Introduction

Schizophrenia is a psychiatric disorder that affects both cognition and behavior. The symptoms of schizophrenia are generally grouped into positive, negative, and cognitive symptoms including hallucinations, delusions, anhedonia, and reductions in attention and memory. The onset of schizophrenia typically occurs in late adolescence to early adulthood (Jablensky, 2000). However, schizophrenia symptoms can manifest differently for each individual and can vary in severity over that individual’s lifetime (Freedman, 2003). Certain genetic variations, stressful life circumstances, and altered brain structure or functions have been linked to a higher incidence of schizophrenia. Nevertheless, both the etiology and the pathology of schizophrenia remain elusive. No single gene is responsible for the development of the disorder and there are no biomarkers to aid in diagnosing patients. These limitations hinder our ability to both diagnose and properly treat schizophrenia. Antipsychotic drugs are typically prescribed to treat the symptoms of schizophrenia and have been a useful therapeutic. These drugs can help alleviate the positive symptoms, and to a lesser extent the negative symptoms of the disorder, but are ineffective in treating cognitive symptoms (Freedman, 2003). In addition, many of these drugs come with undesired side effects like movement disorders (Mentzel et al., 2017) and weight gain (Volavka et al., 2002); which can lead to inconsistent usage and reduced treatment effectiveness (Kane et al., 2013). Therefore, further investigation of the etiology and pathophysiology of schizophrenia can facilitate our understanding of this disorder and is critical for the development of more effective treatments. Several genes which show promise in helping us decipher the developmental risks and mechanisms behind schizophrenia belong to the 14-3-3 family.

The 14-3-3 family of proteins has been identified in all eukaryotic organisms (van Hemert et al., 2001). There are seven known mammalian 14-3-3 genes, each of which expresses a distinct protein isoform; beta (β), gamma (γ), epsilon (ε), zeta (ζ), eta (η), theta (θ), and sigma (σ) (Berg et al., 2003). The highest concentration of 14-3-3 proteins is found in the brain, where they encompass around 1% of the total soluble proteins (Boston et al., 1982). The crystalline structure of 14-3-3 shows that two L shaped monomers come together in a dimerized pair to form a cup-shaped structure (Liu et al., 1995). All 14-3-3 isoforms can form either hetero or homodimers (Takahashi, 2003). This allows for the binding of two regions of the same interacting protein (Berg et al., 2003) or the binding of two different ligands (Fu et al., 2000). Thus, 14-3-3 proteins have a diverse range of hundreds of binding partners. Inside the concave face of the 14-3-3 dimer, polar-charged and hydrophobic amino acids create an amphipathic groove that interacts with phosphoserine and phosphothreonine containing motifs located on its binding partners (Yaffe et al., 1997; Wang et al., 1998). Consequently, 14-3-3 can modulate the function or subcellular location of its binding partners through phosphorylation-dependent protein–protein interactions. Many cellular processes and pathways have been linked to 14-3-3 function and expression. In the nervous system, published studies have indicated the involvement of 14-3-3 proteins in intracellular signaling, cell division and differentiation, apoptosis, and ion channel function (Berg et al., 2003). In addition, both human and animal studies have implicated 14-3-3 in several neurodegenerative and psychiatric diseases, including schizophrenia (Foote and Zhou, 2012).

Several human genome-wide association studies (GWAS) have revealed a genetic link between the 14-3-3 family and schizophrenia. In addition, 14-3-3 knockout animal models have shown schizophrenia-like phenotypes, providing further evidence for this connection. This review will discuss the results of some of the genetic and animal model studies that provide evidence for the link between 14-3-3 and schizophrenia.

## 14-3-3 and Schizophrenia in Human Studies

Genetic linkage and proteomic studies have sought to identify gene or protein expression that may be altered in individuals who are affected by schizophrenia. However, the genetics of the disorder can be heterogeneous; no single gene nor mutation has been identified as the sole cause of schizophrenia. Nevertheless, the 14-3-3 family has been implicated in several studies of schizophrenia patients (Table 1). These results suggest that 14-3-3 alterations may contribute to the development of the disorder; therefore, further investigation of this relationship is warranted.

**TABLE 1:** 14-3-3 Human Studies.

Population/patient sample	Analysis method	Results	References
24 Drug-naïve first-episode schizophrenia patients and 24 age/gender matched control subjects	Expression analysis of peripheral leukocytes	Positive correlation between isoform specific mRNA/protein expression and schizophrenia, negative correlation between ε, θ and ζ expression and the positive symptoms of schizophrenia	Qing et al., 2016
Tissue from 10 subjects with schizophrenia and 11 matched control subjects	DNA microarray analysis of the PFC, ISH analysis and multivariate analysis of covariance	Reduced expression of the β, ζ, γ, and η isoforms in schizophrenia samples	Middleton et al., 2005
1,429 schizophrenia and 1,728 control subjects from a Japanese population	Screened for DISC1-interacting molecules, assessing a total of 25 tagging SNPs	A significant difference in the rs28365859 SNP of YWHAE between schizophrenia patients and controls that corresponded to differential protein expression	Ikeda et al., 2008
72 Schizophrenia patients and 86 healthy controls from a Japanese population	Voxel-based MRI study of the relationship between the YWHAE polymorphism rs28365859 and the OFC subtypes of the “H-shaped” sulcus	rs28365859 SNP of the YWHAE related to changes in the orbitofrontal sulcogyral pattern of schizophrenia patients	Takahashi et al., 2014
186 Control and 188 schizophrenia subjects from 11 studies	Review of proteomic investigations of the brain of schizophrenia patients	Differential expression of YWHAZ and YWHAH, which are located chromosomally close to loci that are disrupted in schizophrenia	English et al., 2011
18 Studies with 1,789 subjects total	Meta-analysis of linkage data applied to published genome scans of schizophrenia	Susceptibility for schizophrenia has been identified on 8p and 22q, YWHAZ located at 8p23, YWHAH located at 22q12.3	Badner and Gershon, 2002
A Northern Chinese population	An association study between three SNPs in the 14-3-3 family and paranoid schizophrenia	SNP rs983583 G/A in the YWHAZ gene showed significant association with paranoid schizophrenia	Jia et al., 2004
35 Schizophrenia, 35 bipolar disorder and 35 control subjects	q-PCR used to determine relative mRNA levels in dorsolateral PFC samples	No significant differences in 14-3-3 mRNA expression levels, a significant genetic association with schizophrenia and SNPS of the ζ isoform	Wong et al., 2005
118 Schizophrenia patients and 118 healthy controls	Investigated allele frequencies of a VNTR in the 5′-non-coding region of the 14-3-3η chain gene	Frequencies of the two-repeat allele were increased in the schizophrenia patients, particularly in those with onset before age 22	Toyooka et al., 1999
1,211 Subjects from 318 nuclear families	A family based genetic association test between subtypes of bipolar disorder	The rs2246704 SNP of YWHAH was associated psychotic bipolar disorder	Grover et al., 2009
18 Control and 18 schizophrenia subjects	Used cDNA to investigate gene expression patterns in several brain regions	14-3-3η differentially expressed in schizophrenia	Vawter et al., 2001
9 Schizophrenia patients and 9 non-psychiatric controls were sourced from the NSW Tissue Resource Center	Alignment of RNA-Seq data to a reference genome and assembled into transcripts for quantification of exons, splice variants and alternative promoters in postmortem superior temporal gyrus	YWHAH and YWHAE differentially expressed	Wu et al., 2012
82 subjects across 7 studies	Review	A deletion in 22q11.2 leads to 22q11.2 Deletion Syndrome, a phenotype that often includes schizophrenia	Bassett and Chow, 2008
1,140 Unrelated schizophrenia cases and 1,140 controls from the Chinese Han population	A genetic association analysis between common SNPs of YWHAE and psychiatric diseases	No association between YWHAE SNPs and schizophrenia	Liu et al., 2011
24 Schizophrenia patients and 24 controls, 308 schizophrenia patients and 135 controls	Systematic search for nucleotide variants in the coding region, 5′ and 3′ untranslated region, and in the exon-intron boundaries of YWHAH	Failed to find significant associations between YWHAH and schizophrenia	Hayakawa et al., 1998
5 cDNAs and 72 ESTs, 21,155 bp of sequence	Systematic screening of YWHAE for polymorphisms in parallel with single-stranded conformational polymorphism analysis	Failed to find significant associations between YWHAH and schizophrenia	Bell et al., 2000
235 Trios: healthy parents and their affected offspring from a Chinese Han population	A family-based genotype association analysis	Failed to find significant associations between YWHAH and schizophrenia	Duan et al., 2005
583 Cases and 372 controls in the Chinese Han population	Investigated several published polymorphisms in the YWHAH gene	Failed to find significant associations between YWHAH and schizophrenia	Wang et al., 2005
52 Controls and 22 schizophrenia patient Caucasian subjects	Immunoreactivity values of cytosolic 14-3-3β and 14-3-3ζ proteins were evaluated by Western blot in the prefrontal cortex	When all schizophrenia subjects were grouped together, no differences in 14-3-3 immunoreactivity were found, but when more appropriately grouped the results did show genetic linkage	Rivero et al., 2015
Japanese sample of 72 schizophrenia patients and 86 healthy controls	Whole brain voxel-based morphometric MRI study regarding the effects of YWHAE SNPs (rs28365859, rs11655548, and rs9393) on gray matter volume	Significant genotype-by-diagnosis interaction for rs28365859 in the left insula, right putamen, and right hippocampus	Kido et al., 2014
Publicly available sequencing data for 6,135 schizophrenia and 9,090 control samples of a European population	Study of the contribution of common and rare risk variants in 14-3-3 genes using ASD and schizophrenia transcriptomic data	Common variants in YWHAE contribute to schizophrenia whereas ultra-rare variants were found enriched in schizophrenia for YWHAZ	Torrico et al., 2020
Brain tissue from 20 schizophrenia cases, 20 bipolar disorder cases, and 20 healthy controls	Used data from comprehensive difference-in-gel electrophoresis (2-D DIGE) investigations of postmortem human hippocampus for Ingenuity Pathway Analysis (IPA) of implicated protein networks and pathways	IPA most prominently implicated 14-3-3 and aryl hydrocarbon receptor signaling in schizophrenia	Schubert et al., 2015
92 young individuals at ultra-high risk for psychosis	Explored the peripheral-blood expression level of the seven YWHA genes using multiplex quantitative PCR	Converters had a significantly higher baseline expression levels for 5 YWHA family genes, and significantly different longitudinal changes in the expression of YWHAE, YWHAG, YWHAH, YWHAS, and YWAHZ	Demars et al., 2020
168 Schizophrenia probands and their families	Genetic association between schizophrenia and the 14-3-3η gene and SNAP-25 genes was analyzed	Significant association with schizophrenia for two polymorphisms in the 14-3-3η gene: a 7 bp VNTR in the 5′ non-coding region and a 3′ untranslated region SNP	Wong et al., 2003

A proteomic pathway analysis revealed that changes in the hippocampus of schizophrenia patients prominently implicate 14-3-3 signaling (Schubert et al., 2015). Further, several studies have revealed that there are 14-3-3 isoform specific changes associated with schizophrenia. In an expression analysis of peripheral leukocytes of drug-naïve first-episode schizophrenia patients, there were four down and one upregulated 14-3-3 mRNA isoforms, and five downregulated protein isoforms (Qing et al., 2016). There was a positive correlation between these isoform specific expression changes and schizophrenia. In addition, there was a negative correlation between the expression of the ε, θ and ζ isoforms and the positive symptoms of schizophrenia. While in a more recent study of peripheral blood expression levels, five of the seven 14-3-3 family members showed significantly higher baseline expression and significant changes in expression in schizophrenia patients who converted to psychosis compared to those that did not convert (Demars et al., 2020). Interestingly, there also is evidence that the 14-3-3 isoform expression levels respond differentially to antipsychotic treatment (Middleton et al., 2005; Rivero et al., 2015). Together these findings suggest that each of the 14-3-3 isoforms may be involved in schizophrenia in different capacities and are worthy of individual investigation. In fact, the genetic link between schizophrenia and individual isoforms has been further studied, particularly the ε, η, and ζ isoforms.

The 14-3-3ε isoform is encoded by the YWHAE gene, which has been proposed to be a schizophrenia susceptibility gene. Gene-based analyses have shown that common variants in the YWHAE gene contribute to schizophrenia (Torrico et al., 2020). In the study of one Japanese population, the rs28365859 single nucleotide polymorphism (SNP) of the YWHAE gene showed a significant difference between schizophrenia patients and controls (Ikeda et al., 2008). The minor allele was more frequent in controls and corresponded to higher protein expression, indicating that a major allele may be a risk factor for schizophrenia. In subsequent MRI studies, the same SNP as well as several others were shown to be related to changes in the orbitofrontal sulcogyral pattern and changes in the volume of the insula, putamen, and hippocampus of schizophrenia patients, possible developmental abnormalities that could contribute to the disorder (Kido et al., 2014; Takahashi et al., 2014).

Among other chromosomal loci, susceptibility for schizophrenia has been identified at the 8p and 22q locations (Badner and Gershon, 2002). 14-3-3ζ is genetically encoded by the YWHAZ gene at the 8p23 location. In addition to being located on a susceptible locus, genetic studies have releveled associations between SNPs and ultra-rare variants of the YWHAZ gene and schizophrenia (Jia et al., 2004; Wong et al., 2005; Torrico et al., 2020). The YWHAH gene which encodes 14-3-3η is located at 22q12.3, another susceptibility locus. Interestingly, a deletion in 22q11.2 leads to 22q11.2 Deletion Syndrome and a phenotype that often includes schizophrenia (Bassett and Chow, 2008). An estimated 1% of schizophrenia patients have also been diagnosed with 22q11.2 Deletion Syndrome. The proximity of 22q11.2 to the YWHAH gene and the overlapping association with schizophrenia seems to indicate a strong genetic link between 14-3-3η and schizophrenia. In further support of this link, both SNPs and variable number tandem repeats (VNTRs) in YWHAH have been associated with schizophrenia in genetic microarray studies (Toyooka et al., 1999; Wong et al., 2003; Grover et al., 2009). In addition, several studies have shown that the 14-3-3η protein is differentially expressed in schizophrenia (Vawter et al., 2001; Altar et al., 2009; Wu et al., 2012). Thus, both YWHAZ and YWHAH genes have been considered as schizophrenia risk genes because they are located chromosomally close to loci that have been genetically associated with schizophrenia (English et al., 2011).

Despite the evidence discussed above for the genetic link between 14-3-3 and schizophrenia, there are some incongruencies in the literature. One study found no association between YWHAE SNPs and schizophrenia (Liu et al., 2011). While several studies have failed to find significant associations between YWHAH and schizophrenia (Hayakawa et al., 1998; Bell et al., 2000; Duan et al., 2005; Wang et al., 2005). In recognition of these discrepancies, Rivero et al. (2015) performed a western blot analysis of schizophrenia subjects and controls. This study indicated that the outcomes of genetic and proteomic studies in schizophrenia are likely influenced by gender, postmortem delay, age, and pharmacological differences in the subjects being tested. When all schizophrenia subjects were grouped together, no differences in 14-3-3 immunoreactivity were found in comparison to controls. However, when the subjects were more appropriately grouped, the results did show genetic linkages (Rivero et al., 2015). While the symptoms, demographics, and environmental factors of schizophrenia are widely varied, it is also important to consider the genetic heterogeneity of the disorder. Therefore, studying the genetic link between just one family of proteins with schizophrenia is further complicated by the fact that over 200 genetic loci have been identified in association with schizophrenia (Legge et al., 2021), and that interaction between two or more of these loci may contribute to the development of the disorder. These data suggest that the heterogeneity of schizophrenia may contribute to mixed results in the literature, especially when there is not proper grouping or when controls are not careful case matched. Thus, it is important to consider these limitations when interpreting genetic linkage studies and their discrepancies. Taking these considerations into account in future work will help further clarify the genetic link between the 14-3-3 family and schizophrenia.

Considering the evidence that 14-3-3 protein expressions are changed in the brain of schizophrenia patients, investigation of 14-3-3 levels in the cerebrospinal fluid (CSF) may serve as a promising new direction to take in the diagnosis of schizophrenia. Although the CSF is an indirect representation of neurochemistry, changes in mRNA and protein levels of various 14-3-3 isoforms could potentially be reflected in the CSF of schizophrenia patients. The 14-3-3 family has been implicated in several other neurodegenerative, neurodevelopmental, and neuropsychiatric disorders (Foote and Zhou, 2012). In fact, CSF levels of 14-3-3 have been used as a biomarker for several of these neurological diseases (Van Everbroeck et al., 2005; Antonell et al., 2020; Figgie and Appleby, 2021; Nilsson et al., 2021) as well as several other diseases (Neal and Yu, 2010; Zeng and Tan, 2018; Morales et al., 2012). Early diagnosis of schizophrenia is difficult, yet early treatment can often make a difference in the prognosis of disease progression and severity. The history of 14-3-3 being used as a biomarker suggests that it could potentially serve as an indicator of schizophrenia, thus further study into this diagnostic possibility is warranted.

## 14-3-3 Animal Models and Schizophrenia

Although human studies have provided valuable insight into the link between the 14-3-3 family and schizophrenia, they do not provide sufficient information about the role of 14-3-3 in the pathogenesis of schizophrenia. In order to better understand the pathology of schizophrenia and how 14-3-3 proteins may be involved; animal models are needed. Several animal models have been used to study the 14-3-3 family in the context of schizophrenia and have provided further support for the link suggested by human studies (Table 2).

**TABLE 2:** 14-3-3 Animal Models.

Mouse strain/line Partial/full knockout Age studied	Behavioral changes	Molecular/synaptic/anatomical changes	References
**14-3-3ζ**			
SV129/14-3-3 ζGt (OST062) Lex Homozygous 5–40 weeks	Hyperactive, lowered anxiety, impaired recognition memory, defect in spatial working memory, defects in sensorimotor gating	Abnormal mossy fiber navigation and glutamatergic synapse formation, 14-3-3 binding to DISC1	Cheah et al., 2012
SV129/14-3-3 ζGt (OST062) Lex Homozygous 30, 35 weeks	Hyperactivity rescued by clozapine, hypersensitive to amphetamine	TH preserved, reduced and mis-localized DAT	Ramshaw et al., 2013
BALB/c/14-3-3 ζGt (OST062) Lex Homozygous 12 weeks, 28–35 weeks	No hyperactivity, no anxiety changes, subtle learning problems, impaired spatial memory	Mis-localized hippocampal cells with aberrant connectivity, reduced spine density, normal DA signaling	Xu et al., 2015
SV129/14-3-3 ζGt (OST062) Lex Homozygous 28–32 weeks	Clozapine affects despair behavior in KOs, anxiety-like behavior not affected by KO or clozapine, clozapine had opposing affects in KO and WT in the Y maze	Dendritic spine defects in the hippocampus and cortex, anatomical differences not rescued by clozapine	Jaehne et al., 2015
**14-3-3ε**			
Mixed (129/S6 and NIH Black Swiss) Heterozygous/homozygous E 18.5	/	Hippocampal defects, cortical thinning, neuronal migration defects, mis-localization of NDEL/LIS1	Toyo-oka et al., 2003
Mixed (129/S6 and NIH Black Swiss) Heterozygous 9–10 weeks	Weak defect in working memory, moderately enhanced anxiety like behavior	/	Ikeda et al., 2008
Mixed (129/S6 and NIH Black Swiss) Heterozygous 12–15 weeks	/	Increased VMAT2 expression in the hippocampus	Iritani et al., 2010
Mixed (129/S6 and NIH Black Swiss) Heterozygous 15 weeks	/	Serpentine, thin, short TH immunopositive fibers, few and sparse dendritic spine like immunopositive varices, significant decrease in TH positive fibers	Sekiguchi et al., 2011
Mixed (129SVE and C57BL/6) Heterozygous/homozygous 12–16 weeks	Hyperactivity, decreased working memory, increased sociability	/	Wachi et al., 2017
**14-3-3ζ/ε**			
129/SvEv Heterozygous E15.5-18.5	/	Abnormal proliferation/differentiation of neuronal progenitors in culture, increased differentiation into neurons, cortical neuronal migration defects, abnormal activity of the Rho family and its effectors	Toyo-oka et al., 2014
**14-3-3γ**			
129SV Heterozygous/homozygous	Normal cage behavior	Several differentially expressed proteins, normal anatomy	Steinacker et al., 2005
ICR outbred *In utero* electroporation of shRNA inhibitor E17-18.5, P3-15	/	Neuronal migration delay of cerebral pyramidal neurons, thicker/highly branched leading processes, impaired ability of the leading process to enter the MZ	Wachi et al., 2016
C57BL/6/B6; CBYwhagGt (pU-21W)266Card Heterozygous 10 weeks	Hyperactivity, depressive like behavior, sensitive to acute stress	/	Kim et al., 2019
**14-3-3 FKO**			
C57BL/6 Transgenic expressed inhibitor 12–24 weeks	Deficit in associative learning and memory	Defects in hippocampal LTP, reduced synaptic NMDARs	Qiao et al., 2014
C57BL/6 Transgenic expressed inhibitor 12–24 weeks	Hyperactivity, unaltered anxiety response, deficit in working memory, deficit in sensorimotor gating, social withdrawal	Cortical neurotransmission deficit, morphological alterations, reduced phospho- cofilin, increase delta catenin	Foote et al., 2015
C57BL/6 AAV delivered shRNA 12–24 weeks	Behaviors recapitulated through acute 14-3-3 inhibition in the PFC and HPC	/	Graham et al., 2019
C57BL/6 Transgenic expressed inhibitor 12–24 weeks	/	Altered neural oscillations in theta/gamma frequency ranges	Jones et al., 2021
B6.SJLSlc6a3tm 1.1 (cre) Bkmn/J B6.Cg-Tg (Camk2a-cre) T29-1Stl/J AAV delivered YFP-difopein 12–24 weeks	/	Increased activation of LS neurons is necessary for over-activation of DA neurons and psychomotor behavior induced by 14-3-3 inhibition in the dCA1	Zhang et al., 2022

### 14-3-3ζ Knockout Mice

One isoform of particular interest in regards to animal models of schizophrenia is 14-3-3ζ. Several 14-3-3ζ knockout models exhibit schizophrenia-like phenotypes. Homozygous 14-3-3ζ knockout mice of the Sv/129 background display behavioral abnormalities including hyperactivity, impaired recognition memory, reduced anxiety, dysfunction in hippocampal-dependent memory, and altered sensorimotor gating (Cheah et al., 2012). It is important to note that 14-3-3ζ knockout in this model is prominent in the hippocampus and dentate gyrus, pointing to the role of these regions in schizophrenia-like behavior. Defects in the hippocampus that may underlie these behavioral changes were apparent before the region was fully developed. The defects included neuronal migration defects, abnormal mossy fiber navigation, and altered glutamatergic synapse formation (Cheah et al., 2012). While the glutamate system may be altered, there is also evidence that 14-3-3ζ knockout affects the dopamine system within this model. The hyperactivity of 14-3-3ζ knockout mice is rescued by clozapine administration and knockout animals are more sensitive to amphetamine administration when compared to wildtype controls (Ramshaw et al., 2013). The mechanisms of action of these drugs and their effects on 14-3-3ζ knockout animals suggest that the dopamine system may underlie some of the behavioral abnormalities in this model. In fact, 14-3-3ζ was shown to have a physical association with the dopamine transporter (DAT). Further, decreased DAT levels found as a result of 14-3-3ζ knockout also led to increased striatal dopamine (Ramshaw et al., 2013). However, clozapine administration was not able to rescue altered anxiety behavior, the structural abnormalities in spine formation, or neuronal mis-localization in 14-3-3ζ knockout mice (Jaehne et al., 2015). This result suggests that the loss of 14-3-3ζ causes changes beyond the dopamine system as well.

Although these studies provide evidence that alterations in 14-3-3ζ can lead to schizophrenia-like phenotypes, these abnormalities have not been fully recapitulated in 14-3-3ζ knockout mice from other genetic backgrounds. When backcrossed into a BALB/c background, homozygous 14-3-3ζ knockout mice can live to adulthood. However, these mice show only weak learning disability and do not differ from controls in many of the same behavioral tests that Sv/129 14-3-3ζ knockout animals display schizophrenia-like behavior (Xu et al., 2015). In addition, the dopamine system appears intact in this model, as dopamine signaling and DAT expression are unaltered in the 14-3-3ζ knockout mice. Despite the discrepancies in behavior and dopamine function, BALB/c homozygous 14-3-3ζ knockout mice do display many of the same structural abnormalities in the brain as 14-3-3ζ knockout mice from the Sv/129 background. These abnormalities included mis-localized hippocampal neurons, reduced CA3 spine density, and abhorrent mossy fiber tracts (Xu et al., 2015).

Interestingly, overexpression of 14-3-3ζ can lead to increased spine density in primary hippocampal neuron culture (Angrand et al., 2006). This is consistent with the reduction in spine density seen in 14-3-3ζ knockout mice, indicating that 14-3-3ζ positively regulates spine density. Overall there is evidence that 14-3-3ζ knockout does induce schizophrenia-like defects in mice, but these defects seem to depend on the genetic background of the animal models. Thus, the models discussed above provide further support for the importance of 14-3-3ζ to many structural processes in the brain that may underlie schizophrenia-like phenotypes when altered.

### 14-3-3ε Knockout Mice

Several human studies have found a link between 14-3-3ε and schizophrenia, prompting further studies in 14-3-3ε knockout animals. Homozygous knockout of 14-3-3ε is prenatally lethal in in-bred genetic backgrounds. Thus, Toyo-oka et al. (2003) examined the brains of homozygous and heterozygous 14-3-3ε knockout mice at embryonic day 18.5, prior to homozygous lethality. Both genotypes had hippocampal defects and cortical thinning, structural issues which were underscored by shortened neuronal migration and mis-localization of key proteins involved in migration processes. The lethality of homozygous knockout mice points to the increased severity of these defects with complete loss of 14-3-3ε. Additional studies of heterozygous 14-3-3ε knockout mice revealed further molecular, structural, and behavioral alterations. One such alteration seen in the hippocampal formation was significantly increased levels of VMAT2, a protein involved in the transport of monoamine neurotransmitters into neuronal vesicles (Iritani et al., 2010). Another study found that 14-3-3ε knockout mice have decreased numbers of tyrosine hydroxylase positive fibers that also exhibit altered functional structure (Sekiguchi et al., 2011). Behavioral testing of this heterozygous 14-3-3ε knockout model revealed weak deficits in working memory and moderately enhanced anxiety like behavior (Ikeda et al., 2008). However, a mixed genetic background model of 14-3-3ε knockout showed different behavioral results, including weaker motor activity, hyperactivity, visual/spatial memory defects, and unaltered anxiety-like behavior (Wachi et al., 2017). Thus, the above evidence suggests that loss of 14-3-3ε causes both structural and behavioral abnormalities in mice that resemble those seen in schizophrenia patient populations. However, these results may be affected by the genetic background of the mouse models in use. Nonetheless, 14-3-3ε knockout models may be a valuable tool in studying the molecular, structural, and behavioral aspects of schizophrenia.

### 14-3-3ζ/ε Double Knockout Mice

Both 14-3-3ζ and 14-3-3ε are critical proteins when it comes to proper brain development, and there is evidence that loss of either can cause defects similar to those seen in schizophrenia patients. The underpinnings of these changes have been further elucidated through the study of a double knockout mouse model in which mice were heterozygous knockout for one isoform and homozygous knockout for the other 14-3-3 isoform (Ywhae^+/flox^; Ywhaz^KO/KO^ and Ywhae^flox/flox^; Ywhaz^+/KO^) (Toyo-oka et al., 2014). Double knockout mice displayed neuronal differentiation and migration defects as well as seizures. The same phenotypes are seen in single knockout models for these proteins but are more pronounced in double knockout animals. These results point to the critical involvement of 14-3-3ζ and 14-3-3ε proteins in the developing brain, as well as the functional redundancy between isoforms. A critical pathway through which these 14-3-3 proteins can regulate neuronal differentiation is the catenin/Rho GTPase/Limk1/cofilin signaling pathway, where 14-3-3 proteins directly interact with phosphorylated delta-catenin to promote F-actin formation. 14-3-3 double knockout mice were shown to have increased levels of delta-catenin, as well as decreased levels of beta-catenin and alphaN-catenin (Toyo-oka et al., 2014). Deletion of delta-catenin did not rescue neuronal migration abnormalities in double knockout mice; but mutants of the Ndel1 protein were able to do so, indicating that 14-3-3 proteins are also involved in a separate pathway that controls neuronal migration (Toyo-oka et al., 2014). Thus, 14-3-3 proteins are important regulators of several different pathways and the loss of one or more isoforms can detrimentally impact neural development and result in behavioral abnormality.

### 14-3-3γ Knockout Mice

14-3-3γ is particularly enriched in the brain and is typically expressed in the developing cortex. Reduction in the γ isoform of 14-3-3 has yielded mixed outcomes when it comes to behavioral and morphological changes. One study found no obvious behavioral alterations or histological differences in the cortex of either heterozygous or homozygous 14-3-3γ knockout mice (Steinacker et al., 2005). While in another study, depletion of 14-3-3γ through *in utero* electroporation of a specific small hairpin RNA (shRNA) resulted in a delay of neural migration and morphological abnormalities in the cortex (Wachi et al., 2016). In a study of behavior, heterozygous 14-3-3γ knockout mice were hyperactive and more sensitive to acute stress when compared to wildtype littermates, while homozygous 14-3-3γ knockout mice died before birth (Kim et al., 2019). Although there are some conflicting results, there is evidence that loss of 14-3-3γ can cause abnormalities that resemble those found in psychiatric disorders like schizophrenia.

### 14-3-3 Functional Knockout Mice

With the many roles of 14-3-3 proteins in neuronal processes and the genetic evidence linking the proteins to schizophrenia, our lab sought to create a mammalian model to study the synaptic and cognitive functions of the 14-3-3 protein family. Transgenic 14-3-3 functional knock-out (FKO) mice were generated through the expression of yellow fluorescent protein (YFP) fused difopein (dimeric 14-3-3 peptide inhibitor), which inhibits all isoforms of 14-3-3 from interacting with endogenous binding partners (Qiao et al., 2014). An important consideration is that 14-3-3 inhibition during embryonic development can be lethal. To avoid prenatal lethality, the transgenic expression of YFP-difopein was driven by the neuronal specific Thy-1 promotor, which is normally expressed in the perinatal period. The Thy-1 promotor created several founder mice in which the expression pattern of YFP-difopein varied but was preserved within the founder line. One of these founder lines had transgene expression that was relatively higher in the hippocampus (HPC) and the pre-frontal cortex (PFC). This line was found to display several behavioral, electrophysiological, and molecular abnormalities. During the contextual fear conditioning and passive avoidance tests, these 14-3-3FKO mice displayed significantly reduced freezing behavior and reduced latency to dark chamber, indicating impairments in associative learning and memory. Electrophysiological investigation of these mice reveled that they also exhibit defects in long-term synaptic plasticity of the hippocampus. Consistently, evidence for NMDAR dysfunction in the 14-3-3FKO line was observed, including significant reductions in the NMDAR/AMPAR ratio and in NMDAR mediated currents, as well as lowered levels of the GluN1 and GluN2a NMDA receptor subunits.

Further investigation of the 14-3-3FKO line revealed additional behavioral and synaptic defects that can be considered schizophrenia-like phenotypes. Increased activity in the open field test (OFT), decreased alteration in the Y maze test, decreased pre-pulse inhibition percentage, and decreased social interaction in the three-chamber test were observed in FKO mice vs. their wildtype (WT) littermates (Foote et al., 2015). These behavioral outcomes reveal deficits in psychomotor behavior, working memory, sensorimotor gating control, and social behavior respectively; all of which can be likened to schizophrenia-related phenotypes. In addition to these behavioral changes, whole-cell voltage-clamp recording of YFP-difopein infected cells in cortical neurons of the FKO mice show significant reductions in the frequencies of spontaneous excitatory and inhibitory post synaptic potentials, as well as alterations in miniature excitatory and inhibitory post synaptic potentials. Further, the cortical layer-5 and hippocampal CA1 pyramidal neurons of FKO mice had decreased distal apical dendrite complexity and decreased spine density when compared to cells from WT littermates. Potential molecular mechanisms of these changes may come from reduced levels of phospho-cofilin and increased levels of delta-catenin in FKO brain tissue.

The behavioral, electrophysiological, and molecular results discussed above indicate that 14-3-3 inhibition in the PFC and HPC can lead to a variety of schizophrenia-like phenotypes. However, the individual roles for each of these brain regions was not distinguishable. In order to determine if inhibition in either the PFC and/or the HPC is necessary and sufficient to induce schizophrenia-like phenotypes, 14-3-3 function was regionally restored through an adeno-associated virus (AAV) delivered shRNA that knocks down YFP-difopein (Graham et al., 2019). Delivery of the shRNA to both the PFC and the HPC lead to significant reductions in OFT locomotor activity, while delivery to one region alone did not. Interestingly, shRNA injection to the HPC alone significantly increased the GluN1 levels in 14-3-3FKO animals, but not to the level of WT littermates. This was likely due to the fact that shRNA was not able to fully inhibit the YFP-difopein transgene. Due to this incomplete inhibition and the fact that the difopein expression in FKO mice is not strictly limited to the PFC and HPC, our lab sought to investigate the effect of region specific difopein expression. A virus using the CamKIIa promotor to drive YFP-difopein expression in excitatory neurons was created to determine if 14-3-3 inhibition in the PFC and/or the HPC is sufficient to induce schizophrenia related phenotypes (Graham et al., 2019). Behavioral testing revealed that WT mice with YFP-difopein injections to the HPC alone, but not the PFC alone, exhibit significantly less freezing behavior in the contextual fear conditioning test and decreased pre-pulse inhibition. In addition, injection of YFP-difopein to both the PFC and HPC, as well as the HPC alone, lead to increased locomotor activity in the OFT, while injection to the PFC alone did not. These results indicate that 14-3-3 inhibition in the HPC is sufficient to induce schizophrenia-like behaviors. In further support of this claim is the finding that YFP-difopein injection to the HPC has a direct effect on NMDA receptor regulation, resulting in decreased GluN1, GluN2A, and PSD95 levels.

To build upon the behavioral and molecular abnormalities found in our models of 14-3-3 inhibition, our lab investigated the role of 14-3-3 proteins in neural oscillations, which are dysfunctional in schizophrenia patients (Jones et al., 2021). FKO animals exhibited a range of changes in power, coherence, and phase-amplitude coupling in both resting and task-related theta and gamma oscillations. WT animals with 14-3-3 inhibited in the HPC alone showed similar yet distinct changes in these same measures. However, 14-3-3 inhibition to the PFC alone leads to few changes in neural oscillations. Overall, our *in vivo* electrophysiological results indicated that acute 14-3-3 inhibition in the HPC largely disrupts theta oscillations and is sufficient to cause neural oscillation defects in the HPC as well as the PFC.

Interestingly, FKO mice exhibit hyperactive dopamine signaling in the ventral tegmental area (VTA) and some of their altered behavior can be attenuated with antipsychotic administration (Foote et al., 2015). Yet there is no YFP-difopein expression detected in the VTA, indicating that 14-3-3 inhibition in other brain areas has an influence on VTA dopamine signaling. Acute 14-3-3 inhibition in the dorsal HPC (dHPC) alone causes c-Fos expression in the dHPC and increased locomotor activity in the OFT that is responsive to antipsychotics (Zhang et al., 2022). This overexcitation in the dHPC is accompanied by robust c-Fos expression in the VTA, indicating a connection between 14-3-3 inhibition induced dHPC overexcitation, hyperactive dopamine signaling, and hyperlocomotion. However, the neural circuitry underlying this connection is not clear, as the dHPC does not directly communicate with the VTA. Through neural tracing techniques, we found that the lateral septum (LS), which also shows increased c-Fos activity after OFT in dHPC injected mice, has mono-synaptic connections with the dHPC (Zhang et al., 2022). Previous studies have shown that the LS also communicates with the VTA, where its connections act on GABAergic interneurons to disinhibit VTA DA neurons (Vega-Quiroga et al., 2018). We confirmed that VTA projecting LS neurons are activated following OFT in difopein injected mice. Thus, the LS appears to be both activated during 14-3-3 inhibition induced hyperlocomotion and anatomically connected with the dHPC and VTA. To further confirm the role of the LS in this neural circuitry we used chemogenetic Designer Receptors Exclusively Activated by Designer Drugs (DREADDs) to manipulate the activity of the LS in mice. Our results demonstrated that chemogenetic inhibition of the LS attenuates difopein induced hyperlocomotion as well as upregulated DA activity, while chemogenetic activation of LS projecting dHPC neurons elicits hyperlocomotion in WT mice. These results indicate that increased activity in the LS is both necessary and sufficient to induce changes in psycholocomotor behavior. Overall, our results provide evidence for a polysynaptic pathway from the dHPC to the LS to the VTA in which 14-3-3 inhibition causes an imbalance in the ratio of excitatory to inhibitory signaling that results in psychomotor behavior (Zhang et al., 2022).

Through a series of studies, our lab has created a mouse model of 14-3-3 inhibition that can be useful in the study schizophrenia related phenotypes at the behavioral, molecular, and circuitry levels. The family of 14-3-3 proteins is involved in several critical neuronal processes and has been associated with schizophrenia through genetic linkage studies. We found that transgenic expression of a 14-3-3 inhibitor in key forebrain areas results in a mouse line that displays several behavioral, electrophysiological, and molecular phenotypes which resembles those seen in schizophrenia patients. Through acute viral delivery of our inhibitor, we saw that the functional loss of 14-3-3 proteins in the hippocampus is sufficient to induce these disease-related outcomes. Using our 14-3-3 inhibited mice, we delineated a previously unknown polysynaptic circuit that connects 14-3-3 inhibition induced imbalances in neuronal signaling to changes in psychomotor behavior. Together, our work provides further evidence for the role of 14-3-3 dysfunction in schizophrenia and a tool which we can use to further explore the mechanisms of pathology in schizophrenia.

## Potential Mechanisms

Human genetic and proteomic linkage studies have provided several lines of evidence that the 14-3-3 protein family and their associated genes may be altered in schizophrenia. Because the 14-3-3 proteins mediate such a wide range of cellular and molecular processes, any mutation to or change in expression of these proteins may contribute to abnormalities in these processes and potentially to disease states such as schizophrenia.

Several neurotransmitter systems appear to be altered in schizophrenia, including the dopamine, glutamate, and GABA systems. For many years the dopamine hypothesis and the glutamate hypothesis were separate lenses in which researchers and physicians studied and tried to treat the neuropathology of schizophrenia. More recently, it has been proposed that dysfunction in the dopamine system may be a downstream consequence of dysregulated glutamate neurotransmission in the forebrain. As the major excitatory neurotransmitter, glutamate has influence all over the brain, including in the dopamine system. However, it is unknown how the two systems could be interacting in the disease state, making it difficult to merge the two hypotheses for schizophrenia. The 14-3-3 protein family may serve as a potential link between dopamine and glutamate dysfunction in schizophrenia, as there is evidence that changes in 14-3-3 have effects in both neurotransmitter systems. Alterations in NMDA receptor (NMDAR) activity is reported in several 14-3-3 knockout models, and decreased NMDAR activity can alter the excitation and inhibition balance in neural networks and circuits (Moghaddam and Javitt, 2012). Surface expression of NMDA receptors is regulated and promoted by 14-3-3 proteins, particularly the ζ and ε isoforms, through their interactions with particular NMDAR subunits (Chen and Roche, 2009; Lee et al., 2021). This relationship is further highlighted by the finding that knockdown of the NMDAR subunit NR1 leads to synaptic reduction of 14-3-3ε (Ramsey et al., 2011; Ferris et al., 2014). Further, a recent study from our lab has elucidated a polysynaptic pathway in which 14-3-3 dysfunction in the dorsal hippocampus underlies altered psychomotor behavior mediated by dopamine in the VTA (Zhang et al., 2022). These results offer evidence on how the glutamate and dopamine systems may be linked in schizophrenia, as well as a potential role for 14-3-3 dysfunction in the mechanism of the pathology.

The 14-3-3 proteins have many binding partners and are involved in several critical molecular and cellular pathways; thus, the disruption of these proteins can have many potential impacts. One pathway affected by 14-3-3 disruption is the catenin/Rho GTPase/Limk1/cofilin signaling pathway, which plays a significant role in regulating actin cytoskeleton dynamics during brain development. Moreover, the Ndel1/Lis1/14-3-3ε complex has been proposed to be critical for neuronal migration (Foote and Zhou, 2012). Interestingly, the localization of the Ndel1/Lis1/14-3-3ε complex to axons is regulated by the schizophrenia related protein DISC1 (Taya et al., 2007). 14-3-3ε binds to the DISC1 binding region of Ndel1, maintaining its phosphorylation (Toyo-oka et al., 2003; Johnson et al., 2010), and deficiency of 14-3-3ε leads to mis-localization of Ndel1 and Lis1 (Toyo-oka et al., 2003). Additionally, 14-3-3 proteins have been shown to interact with many other cytoskeleton and dendritic spine related proteins. For example, 14-3-3ζ interacts with microtubule-associated protein/microtubule affinity-regulating kinase 3 (MARK3) (Angrand et al., 2006). The proper regulation of microtubules is necessary for neuronal migration and spine formation. In fact, knockout of microtubule associated protein 6 (MAP6) in mice results in many similar schizophrenia-like phenotypes as 14-3-3 knockout models. These include deficits in synaptic plasticity, abnormal glutamatergic signaling, and locomotor hyperactivity (Cuveillier et al., 2021). The similarities between MAP6 and 14-3-3 knockout models along with the physical interaction between 14-3-3 proteins and microtubule related proteins suggest that 14-3-3 are key players in appropriate cytoskeletal regulation. The association between 14-3-3 proteins and these specific binding partners and pathways may underlie the neuronal migration and synaptic defects observed in the 14-3-3 knockout animal models discussed above, as well as provide potential mechanistic insight into the pathogenesis of schizophrenia.

Several of these processes are critical to neurodevelopment, suggesting that any changes in 14-3-3 proteins during critical periods could potentially lead to abnormal structural and functional connections in the brain. In fact, the viability of knockout animals and the severity of their abnormalities in the brain are influenced by the timing of the 14-3-3 knockout. Similarly, the timing of the progression of schizophrenia in humans also points to the importance of these critical developmental periods. The molecular changes that underlie schizophrenia require further study to create a more holistic understanding of the disease and how 14-3-3 dependent regulatory pathways may be involved. The diverse and important roles for 14-3-3 proteins serve as promising points from which to study the mechanisms underlying the disorder.

## Conclusion

Schizophrenia is a complicated mental disorder that greatly affects those who are diagnosed with it. The heterogeneity of the disorder makes it difficult to decipher the neurobiological basis of schizophrenia. One interesting and promising route of study in schizophrenia is the role of 14-3-3 proteins. The genetic link between 14-3-3 and schizophrenia suggests that studying schizophrenia through the 14-3-3 family can offer valuable insight to the disease. The results from 14-3-3 knockout models validate this idea and have allowed for a better understanding of the pathology of schizophrenia and how the 14-3-3 family may contribute to its progression. The 14-3-3 protein family has a wide variety of binding partners and functions, the exact mechanisms behind how alterations in 14-3-3 proteins can lead to schizophrenia-like phenotypes is not fully understood. However, some potential mechanisms include abnormal neural development and neuronal signaling following altered neurotransmitter receptor levels, mis-localized protein complexes, and interrupted cellular pathways. Future study will be needed to fully elucidate the causes of these observed phenotypes but will undoubtedly provide further understanding of the complex pathology of schizophrenia.

## Author Contributions

MN was responsible for drafting, writing, reviewing, and editing this manuscript. YZ was responsible for writing, reviewing, and editing the manuscript with suggestive ideas on formatting and outlining this manuscript. Both authors contributed to the article and approved the submitted version.

## Conflict of Interest

The authors declare that the research was conducted in the absence of any commercial or financial relationships that could be construed as a potential conflict of interest.

## Publisher’s Note

All claims expressed in this article are solely those of the authors and do not necessarily represent those of their affiliated organizations, or those of the publisher, the editors and the reviewers. Any product that may be evaluated in this article, or claim that may be made by its manufacturer, is not guaranteed or endorsed by the publisher.
